# Computational Chemical Synthesis Analysis and Pathway Design

**DOI:** 10.3389/fchem.2018.00199

**Published:** 2018-06-05

**Authors:** Fan Feng, Luhua Lai, Jianfeng Pei

**Affiliations:** ^1^State Key Laboratory for Structural Chemistry of Unstable and Stable Species, Beijing National Laboratory for Molecular Sciences, College of Chemistry and Molecular Engineering, Peking University, Beijing, China; ^2^Center for Quantitative Biology, Academy for Advanced Interdisciplinary Studies, Peking University, Beijing, China; ^3^Center for Life Sciences, Academy for Advanced Interdisciplinary Studies, Peking University, Beijing, China

**Keywords:** chemical synthesis analysis, retrosynthesis, pathway design, deep learning, seq2seq

## Abstract

With the idea of retrosynthetic analysis, which was raised in the 1960s, chemical synthesis analysis and pathway design have been transformed from a complex problem to a regular process of structural simplification. This review aims to summarize the developments of computer-assisted synthetic analysis and design in recent years, and how machine-learning algorithms contributed to them. LHASA system started the pioneering work of designing semi-empirical reaction modes in computers, with its following rule-based and network-searching work not only expanding the databases, but also building new approaches to indicating reaction rules. Programs like ARChem Route Designer replaced hand-coded reaction modes with automatically-extracted rules, and programs like Chematica changed traditional designing into network searching. Afterward, with the help of machine learning, two-step models which combine reaction rules and statistical methods became the main stream. Recently, fully data-driven learning methods using deep neural networks which even do not require any prior knowledge, were applied into this field. Up to now, however, these methods still cannot replace experienced human organic chemists due to their relatively low accuracies. Future new algorithms with the aid of powerful computational hardware will make this topic promising and with good prospects.

## Introduction

Although the concept of organic chemistry was proposed before the nineteenth century, the first steps of synthesis analysis took human beings more than 100 years, from 1828, when the German chemist Friedrich Wöhler produced urea with potassium cyanate and ammonium sulfate (Leicester and Klickstein, 1951), to mid-twentieth century, when chemists such as Robinson, Woodward, and Corey raised it to a qualitatively higher level of sophistication with the idea of retrosynthetic analysis (Corey, 1988). Since then, laboratories around the world have made remarkable achievements in total synthesis, biosynthesis and biomimetic synthesis. The standard flow of synthesis pathway planning has made it possible for scientists to design computer programs to deal with synthetic problems.

Since the Dendral Project (although failed) of Stanford University in the 1960s, experts in chemistry, biology and computer science showed great enthusiasm in developing relevant algorithms in the next 30 years, but few breakthroughs were made and more people viewed it as a “mission impossible.” Actually, this task was too complex for scientists at that time when machines could only deal with very simple molecules which humans did not need much assistance with. However, after the 1990s, the developments of new efficient algorithms and more well-designed databases including Reaxys and SciFinder (providing chemists the source of structured data of chemical reactions) lighted the passion for computer-assisted synthesis design again. And more cheminformatics tools were proposed, including the development of molecule descriptors and molecular encoding methods like SMILES (Simplified Molecular Input Line Entry Specification) (Weininger, 1988).

Early retrosynthesis analytic systems were mainly reaction rule-based, such as LHASA (Corey et al., 1972a,b), SYNLMA (Johnson et al., 1989). Different rule-based methods focused on different concepts, including reaction mechanisms, skeletal construction and some classic reactions between common groups. However, rule-based methods cannot cover the whole organic reaction space and probably give out incorrect results (e.g., the algorithms would produce a compound which never exist, or forget to protect groups with high reactivity).

After 1990, many new methods using machine learning as an important tool were proposed, but most of them still followed the concepts of traditional reaction rules. So we define them as “two-step models”—machine learning played the role of decision making, and decision generating were related to reaction rules or structural rules. In recent years, deep learning (or deep neural networks) techniques have been applied in reaction prediction and retrosynthesis analysis. For example, regarding reactions as translation between two languages (“reactants” and “products”), seq2seq (two recurrent neural networks) (Sutskever et al., 2014) was used in synthetic prediction. However, these modern tools still need essential improvements to meet the need of organic chemists. Also, negative samples are quite important in machine learning, but reaction databases seldom provide information about “A do not react with B,” which is a severe limitation.

Recently, in the field of drug design, modern methods have changed the trial-and-error and time-consuming lab work into computational process. After designing molecules according to certain principles, medicinal chemists will have to synthesize the designed molecules. With modern web resources (Khan et al., 2011; Yadav et al., 2016), computers can take the synthesis pathway into consideration. For example, databases like KEGG enzymatic reaction and ChemBioFinder have benefited a lot in both drug discovery and drug synthesis prediction.

Organic reactions are not like the process of chess or Sudoku games, because they are full of exceptions and rarely have fixed rules, so it presents great challenge for computer programs. With the general trend of artificial intelligence (AI), scientists realized the combination of AI and synthetic planning would probably be the general trend in this field. Although we cannot guarantee the correctness of one computer-designed synthetic route, AI may probably come up with incredible new ideas beyond human ones, and its comprehension of complex reaction patterns such as rearrangement and catalytic cycles may be superior to humans, too. To sum up, we believe that computers will help scientists to a great extent in the field of synthetic analysis and pathway design in the future.

## Synthesis prediction with network searching and rule matching

### Building and searching reaction networks

**Figure 1:**
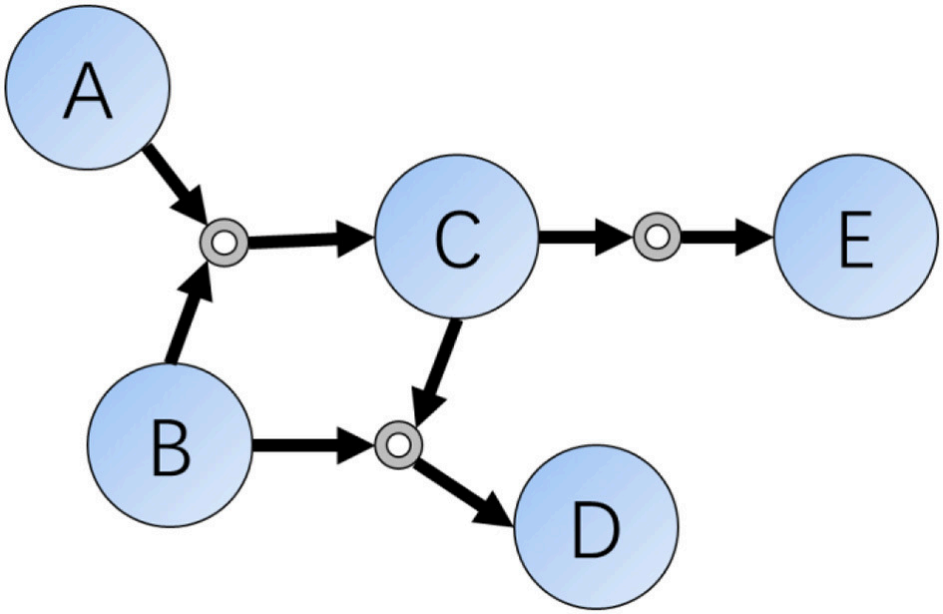
Schematic representation of a local part of the Reaction Networks. Reactions included in this figure are: (1) A + B = C; (2) B + C = D; (3) C = E.

As we all know, one decisive character differing between humans and computers are the ability of memory. For organic chemistry experts, they often memorize hundreds of classic reactions and rules, but modern computers have the ability to store and search for chemical databases as large as the entire set of known molecules and reactions. In a computer scientists' view, chemical reactions are sets of data indicating relationships or connections of compounds, and this kind of existence can be represented as data structures such as connections or networks. According to these ideas, Grzybowski et al. did such a kind of transformation in early 2000s and finally finished the Network of Organic Chemistry (NOC) (Fialkowski et al., 2005; Bishop et al., 2006; Grzybowski et al., 2009), which contains more than ten million organic reactions (edge) connecting a similar number of compounds (vertex) (Figure 1).

The searching process is not simple. Grzybowski's group tried different ways to do global minimization in their program Chematica. They took two factors into consideration: one is the overall “cost” C_tot_ of a pathway (including labor, purification costs, etc.) and the cost of starting materials. The other is the popularity scoring function P_tot_ which prioritized more popular reactions. For the searching algorithm, one approach is to minimize the scoring function in each “depth” of searching and gradually increase the “depth” to produce the synthetic pathway. Traditional BFS (breadth-first-search) (Lee, 1961) is also adapted to synthetic planning to generate many possible pathways. These searching algorithms can simplify the “combination explosion” problems into simple and intuitionistic ones, which can be solved within a few seconds. In addition, due to the specific data structure of NOC, Chematica has the Synthesis Optimization with Constrains (SOCS) scheme, too, which supports the existence of constraints, such as the maximum number of products and avoidance of certain intermediate. This process is just like finding a function's minimum value with constrains. And without doubt, satisfying any constraint factors will probably cause a trade-off of an increased cost function.

### The development of rule-based synthetic design

Although reaction networks can guarantee the validity of predicted retrosynthesis reactions, it was a much difficult task for early pioneers to collect reaction databases as big as NOC. The first ideas of chemists and computer scientists were using reaction rules to predict retrosynthesis reactions, and developing logic-based and knowledge-based searching strategies for designing reaction routes. By applying retrosynthetic (backward generation) procedure which was proposed in the mid-twentieth century, in theory, computers can generate reasonable starting materials and reaction pathways. However, although there are many rules and famous name reactions in the field of organic chemistry, choosing which reaction to use are things that really matters. One of the earliest pioneers, Dendral project (Lindsay et al., 1993) started by a Stanford team did not realize this goal. As one of the contributors of retrosynthesis analysis, Corey raised his rule of breaking bonds and planning the synthetic pathway, which can be taught to computers. Although it is far from mature in today's view, Corey and his idea had raised computer-assisted pathway design to a higher level. In 1969, Corey and Wipke presented the first computer-aided synthesis design software called OCSS for Organic Chemical Simulation of Synthesis (Corey and Wipke, 1969). It was then split into two directions: LHASA (Corey et al., 1972a,b) in Corey's group and SECS (Wipke et al., 1978) developed by Wipke. After that, many followers proposed different kinds of rule-based methods, which were introduced in detail in other recent reviews (Szymkuć and Gajewska, 2016). Here we only briefly list some of them in Table 1.

**Table 1:** Summary of some rule-based retrosynthesis models.

**Model name**	**References**	**Reaction rules**	**Limitations and problems**
LHASA & SECS	Corey et al., 1972a,b; Wipke et al., 1978	Expressing several simple design strategies by a chemical language called CMTRN (ChemistryTRaNslator).	Few reaction rules No stereochemistry Not active for years
SYNLMA	Johnson et al., 1989	Using knowledge base to do logical operations.	The problem of combination explosion
IGOR & IGOR2	Bauer et al., 1985; Ugi et al., 1993	Transforming molecules into bond-electron (BE) matrices & transforming reactions rules were into the subtraction of reactant and product matrices.	High computationally cost
CHIRON	Hanessian et al., 1990	Trying to maximize the overlap between targets and start materials.	CHIRON does not search full synthetic tree and can only be used to assist humans
WODCA	Hollering et al., 2000	Analyzing the characters of bonds to suggest which one should be regarded as the retrosynthetic disconnections with matrix notation.	Slow computational speed
Syntaurus	Szymkuć and Gajewska, 2016	Using 20,000 expert-coded and cross-checked chemical transforms and using CSF (Chemicals' Scoring Function) + RSF (Reaction Scoring Function) to evaluate and rank the synthetic routes.	Many years were taken to construct the database Some reactions are not applicable in real lab work

For rule-based *de novo* synthesis prediction, there exists mainly two challenges. The first one is the collection of reaction rules. Early pioneers like LHASA and SECS are relatively weak in the number and diversity of reaction rules, while later programs like Syntaurus can meet the requirements of basic coverage of reaction space. The other challenge is ranking or scoring of pathways. To deal with this, different synthetic-planning programs used various types of methods ranging from bond disconnections in LHASA to minimize the combined scoring function in Syntaurus.

Perhaps the challenge has been tackled too early, as organic reactions are full of exceptions. Rule-based methods still cannot meet the full requirement of organic chemists. In practice, some relatively rare reactions, paradoxically, can be of vital importance in some particular synthesis, so generalized rules may not be the ample knowledge for computers, instead, some specialized cases are also needed. Moreover, most algorithms cannot predict issues of stereo- and regio-chemistry until the general application of SMILES and SMARTS (which can take these factors into consideration). Limitations of searching space and lack of intelligent algorithms still call on scientists to explore new revolutionary ways to predict synthetic pathways—that is why machine learning was becoming more and more popular in the past decade.

## The application of machine learning in synthetic design

### Automatically learning reaction rules

Manual encoding of organic reaction rules has some obvious disadvantages. Since it relies on the experience of a small number of chemists, it usually did not cover enough fraction of the reaction space and few of them can be as ample as Syntaurus. Moreover, it is not realistic to exhaustively define the full substrate scope and incompatibilities for every possible reaction, and conflicting reactivity is rarely black and white; incompatibility depends on the exact nature of the reacting molecules. These factors motivate the development of an automated approach to the forward reaction evaluation.

**Figure 2:**
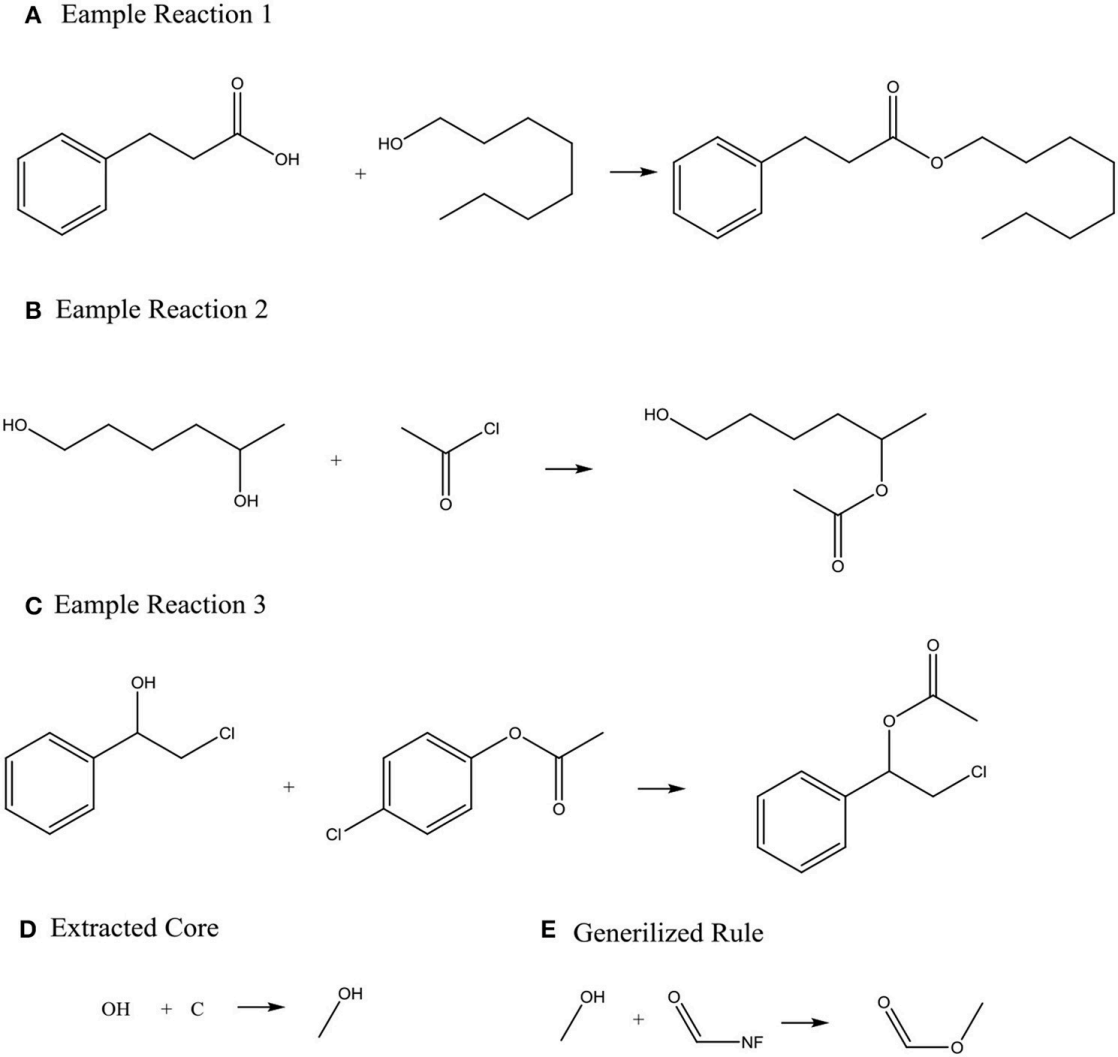
The process of extracting reaction rules. **(A–C)** Identifying the Reaction core (the set of atoms where connections or bonds have changed by going from reactant to product) by comparing reactants and products, and extending the cores to contain neighboring atoms or functional groups. **(D)** Clustering the extracted reaction cores into common groups. **(E)** Producing a generalized rule template for each cluster group and completing the generalized rule templates.

Systems with machine-generated chemistry rules were first published in the early 1990s such as the example SYNCHEM (Gelernter et al., 1990), which also use machine learning to increase its knowledge base. The KOSP (Satoh and Funatsu, 1999) program (Knowledge Base-oriented System for Synthesis Planning) attempts to extract rules from reaction databases by clustering reactions based on characteristics of atoms within three bonds of a disconnection site. Similarly, RETROSYN (Blurock, 1990) also provided an interactive search based on finding single disconnections by similarity with precedent reactions. The system ARChem Route Designer (Law et al., 2009) developed by SymBioSys realized a systematic mode for automatically extract reaction rules and applied these rules in retrosynthetic design. However, it also has the limitation of not accounting for stereochemistry and/or regiochemistry like most rule-based system. Figure 2 illustrates how ARChem Route Designer learns reaction rules from reaction pools.

ARChem Route Designer provides the method to generate synthesis trees. This method still has some weakness. First, the long-distance effect was neglected, for example, the existence of hydroxyl in the distance of several bonds can accelerate leaving of groups such as –OSO_2_CH_3_. Second, some conflicts might happen when there are two or more reactive groups in a molecule. Nevertheless, this approach already proved that computer's ability to learn reaction rules can make it possible for fully data-driving and automatic pathway designing algorithms.

### Two-step models—combination of rule-based model and machine learning

**Figure 3:**
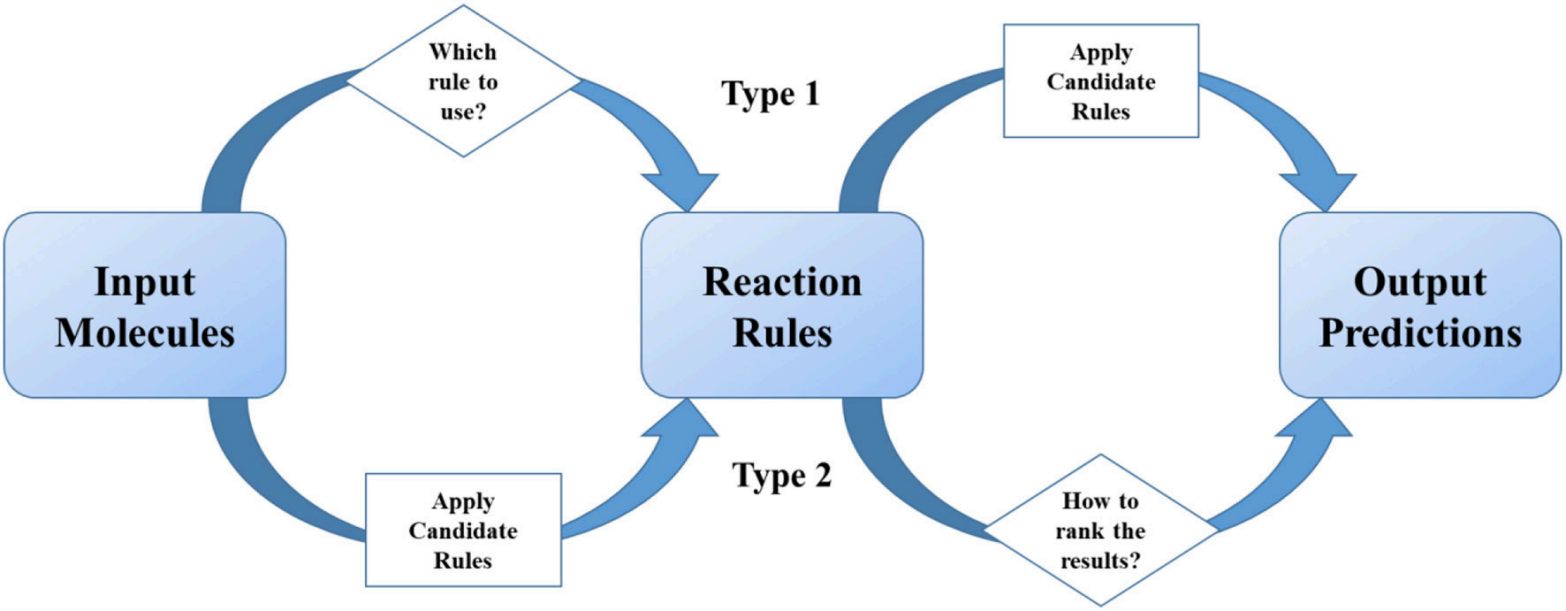
Reaction rules play the intermediate role in two-step models. The judging or ranking (in diamond blocks) is implemented by using machine learning or deep learning methods.

Methods summarized in section The Development of Rule-based Synthetic Design emphasized the importance of reaction rules as traditional organic chemists do. As statistical methods get more and more popular in recent two decades, scientists tried to combine reaction rules with data science skills, especially machine learning. We define these models as two-step ones, which undergoes two separate steps (1) the first step is for providing excess possible reaction results, and the second is for ranking or scoring of them; (2) or the first step is for classification of reactions, and the second is for applying certain pre-coded rules. In a two-step method, “reaction rules” play the role of important intermediates in the models (Figure 3).

SYNCHEM (Gelernter et al., 1990) was one of the earliest effort in the application of machine learning methods to chemical predictions, relied on clustering similar reactions, and learning when reactions could be applied based on the presence of key functional groups. While SYNCHEM uses active node and non-active node to label the molecules, other subsequent machine learning algorithms are based on molecular descriptors to characterize the reactants in order to guess the outcome of the reaction. Such descriptors include information both from experimental/physico-chemical measurements, such as dipole moment, and theoretical/structural information such as the number of rings, to represent the properties of the molecule. With descriptors as the fingerprint of molecules or reactions, computer algorithms become more likely to do classification or similarity calculation. Schneider and collaborators' work (Schneider et al., 2015) is an example to use molecular descriptors to generate reaction fingerprints and classify organic reactions into 50 classes, with methods of random forests, naïve bayes, K-means and logistic regression. If the input is shortened to only include reactant or product, this method can be applied to reaction prediction or pathway design.

During the last 10 years, there were many algorithms published to predict the outcome of organic reactions, which still rely on reaction rules but use machine learning to judge which rule to choose. Although the ideas are similar, they differ in some details. Since outcome prediction is forerunners of retrosynthesis analysis in this field, we briefly introduce some of the relevant algorithms. Carrera et al. used machine learning to predict chemical reactivity of organic molecules (Carrera et al., 2009). They train random forest models for certain molecules (such as BuNH_2_ and NaCNBH_3_) to predict their reactivity. However, it was unlikely to give every compound an independent model, so it was far from a generalized reaction prediction system. The CSB (Chemical Sense Builder) system (Fica and Nowak, 2005) proposed by Fica and Nowak can simulate and predict organic reactions. This system consists of two separate functional modules, which can be used individually or sequentially. The first one contains four logic-based and knowledge-based models for generating and discovering reactions. The second one mainly applies learning tools for reaction simulation process. The CSB takes account of a set of mechanisms controlling the course of reaction generation, even considering thermodynamic concept (reaction enthalpy), and common reactive sites, searching for analogies in reaction database.

Reaction Predictor (Kayala et al., 2011; Kayala and Baldi, 2012) by Kayala et al. is an algorithm that first identifies potential electron sources and electron sinks in the reactant molecules based on atom and bond descriptors. The first component is a proposal model analyzing structures of input molecules and propose all possible reactions according to the mechanism of reactions. Finally, neural networks are used to determine the most likely combinations in order to predict the true mechanism. The reported accuracy is 78.1% for polar reaction, 85.8% for pericyclic reactions and 77% for radical reactions. While this approach allows for the prediction of many reactions at the mechanistic level, many organic chemistry reactions have relatively complicated mechanisms with several elementary, which would be costlier for this algorithm to predict. However, it does not require any reaction template.

Coley et al. also applied the idea of two-step analysis like ReactionPredictor too, but their way of generating the set of possible products is different (Coley et al., 2017). First, they generated a set of chemically plausible products according to pre-inputted reaction rules. During this process, they also mentioned the importance of negative sampling like Segler and Waller, and they expanded existing reaction databases with negative reaction examples. Second, softmax neural network layer (i.e., an exponential activation function that maps a list of numbers to a list of probabilities that sum to one) was applied to generate probabilities of each product. The most creative part was to use “edit-based” information as the feature of learning. Four kinds of information were inputted: (1) An atom a_i_ loses a hydrogen; (2) An atom a_i_ gains a hydrogen; (3) Two atoms, a_i_ and a_j_, lose a connecting bond b_ij_; (4) Two atoms, a_i_ and a_j_, gain a connecting bond b_ij_, and output will be the probability. Combining edit-based model and baseline model (only concern about the structure of products), the hybrid model gives the accuracy of 71.8% for top-1, 86.7% for top-3, and 90.8% for top-5. It can also be applied to predict retrosynthetic reactions.

Wei et al. (2016) used a graph-convolution neural network proposed by Duvenaud et al. (2015) to infer fingerprints of the reactants and reagents, and then predict the outcome of reactions based on reactant fingerprints. This kind of fingerprints were generated from molecule graphs, in which nodes represent atoms and edges represent bonds. At each layer of a convolutional neural network, information flows between neighbors in the graph. Finally, this model will generate a fixed-length fingerprint vector. In the afterward predicting algorithm, Wei et al. classified organic reactions into 16 different types (for alkyl halides and alkenes) and use SMARTS transformation to describe the transformation between product molecules and reactants. This method can achieve an accuracy of 85% of test set reactions and 80% of selected textbook questions from Wade problems (Wade, 2013). In fact, previously developed machine learning algorithms were also able to predict the products of these reactions with similar or better accuracy, but the structure of their algorithms allow for greater flexibility. However, only 16 types of reactions covering a very narrow scope of possible alkyl halide and alkene reactions limits the application of the algorithm. Furthermore, the effect of secondary reactant or reagent was over-simplified as only 50 common ones were taken into consideration.

Segler and Waller built a knowledge graph using reaction templates (Segler and Waller, 2017a), which resembles NOC described in section Building and Searching Reaction Networks. With some additional network-based calculation, this model can find novel reactions by searching for missing nodes in the graph and predict the catalysts of reactions. Although they did not include machine learning then, one major advancement is their idea of negative sampling. As they mentioned, while the positive evaluation of a reaction prediction system can be easily done with a test set of hold-out known reactions, negative evaluation with reactions that are known not to occur is a difficult task, because failed reactions or the limitations of synthetic methodology were seldom published. This lack of data has been criticized both by synthetic chemistry and chemoinformatics community. To get data on reactions which are unlikely to occur, Segler and Waller randomly selected 36,000 known reactions from their validation set and generated “wrong” (but some still plausible) products with hand-coded reaction rules. Then the model can identify the wrong products and label these reactions as unlikely to occur. That means negative samples can be generated by computers, which greatly helped the development of machine learning in the field of reaction prediction.

Although these methods are not designed specifically for retrosynthesis, some of them can be modified to meet the requirements of retrosynthetic prediction, too, such as Segler and Waller's reaction graph, Coley et al.'s ReactionPredictor and Wei et al.'s graph-convolution neural network. These methods, together with other earlier retrosynthesis methods related to machine learning are in common because they all divide the task into two separate steps, they all undergo an intermediate step—reaction rules. Similarly, programs specialized for reaction pathway prediction can also adopt this process. One important work is Segler and Waller's neural-symbolic approach (Segler and Waller, 2017b) for retrosynthesis and reaction prediction, as well as synthetic pathway design. Since it is specially designed for retrosynthesis analysis, it must have some distinguished features—global information has to be considered to avoid conflicts. For example, for carbon-carbon coupling reactions, when there are carboxyl or aldehyde groups in the target molecule, Kumada reaction should be abandoned because the Grignard reagent will react with these groups, so we can only choose Suzuki, which uses R-B(OH)_2_ instead of RMgBr. In their neural-symbolic method, the computer has to learn which named reaction can be used to produce a molecule (or under which rule the starting materials reacted) with all information about the molecule. By training neural networks with millions of examples of known reactions and the corresponding correct reaction rules, computers will give each input a label of reaction type. Their reaction data are from the commercially available Reaxys database. The input information is ECPF4 (Unterthiner et al., 2014) of targeting molecule. Because this fingerprint a fixed-length indicator, a neural network with one hidden layer (Clevert et al., 2015) or a deep highway network can be applied. The neural network on molecular fingerprints to prioritize rules are combined with a Monte Carlo tree search, which can realize the function of retrosynthetic reaction prediction. When applying retrosynthesis prediction several times, we can get the synthesis pathway. Segler and Waller used 103 hand-coded reaction rules, such as Diels-Alder, Sonogashira, Kumada. Their model can predict retrosynthesis reaction rules in an accuracy of 78% (top-1) and 98% (top-3). Then, they replaced 103 hand-coded reaction rules with automatically-extracted 8,720 reaction rules from 4.9 million examples. Although the accuracy decreased to 64% (top-1) and 95% (top-3), this approach is fully end-to-end and data-driving. However, they reported an average of 44.5 matches per query, suggesting the coverage might be not enough.

In Segler et al. (2018) published their updated model. In this work, they proposed a 3N-MCTS approach for chemical synthesis prediction, which means three neural networks combine with Monte Carlo tree search (MCTS). Like their previous work, reactions published in Reaxys before 2015 were used to extract reaction rules (contain the information of reaction center), and two separate neural-symbolic models are trained—relatively slower “expansion policy” for selecting best candidate transformations and faster “rollout policy” for estimating synthesis positions values. Then by generating negative examples as they did in their previous work, a binary filter network for predicting whether reactions really occur were trained, thus every reaction proposed in the expansion process would be evaluated and only feasible ones are kept, which greatly reduced the risk of wrong output. Following the process of selecting, expansion, rollout and update, 3N-MCTS model can give result much more quickly than any other methods such as plain Monte Carlo, and BFS. In double-blind test, even chemists cannot distinguish literature and 3N-MCTS results. However, quantitatively prediction of enantiomerism is still an unsolved problem in this model. Because of the coverage of training set, the accuracies of synthetic prediction for natural products are limited.

For all the methods mentioned in this section, reaction rules are still the most important guidance of reaction prediction and pathway design, and machine learning is more like assisters. The common limitation of this kind of system, as well as other rule-based ones, is that they do not take stereochemistry into account. We are curious if it can be solved with more reaction examples or other descriptors, such as stereo-chemically aware descriptors (Carbonell et al., 2013). But it is indubitably that, machine learning greatly accelerates the development of retrosynthesis design. Although these methods have not fully got rid of the idea of rule-guided design, the wide application range and high accuracy is really impressing.

### Fully end-to-end retrosynthesis analysis with deep neural networks

In recent years, deep neural networks have been applied to this field. One characteristic feature is that computers do not need to follow human-defined reaction rules, and instead, they can re-comprehend chemical reactions with only millions of reaction examples. So we call these methods end-to-end ones—scientists only provide computers with two ends—one end is reactant and the other is product. These methods are fully data-driven. One exception is mentioned in section Two-Step Models—Combination of Rule-Based Model and Machine Learning—the first template-free approach introduced by Kayala et al. (2011) and Kayala and Baldi (2012). Because it can predict a series of mechanistic steps to obtain one reaction outcome using fingerprints and handcrafted features, it was based on common reaction mechanisms, and not fully data-driven. Using end-to-end analysis with deep neural networks, many approaches were proposed in recent years.

In order to implement the end-to-end methods, one kind of approaches is to define some special data structures to help computers understand the concept of reactions. These data structures are far from traditional “reaction rules” which can be understand by human beings. An important example is Jin et al.'s research (Jin et al., 2017) with a novel approach based on Weisfeiler–Lehman Networks (WLN) (Lei et al., 2017). They trained two independent networks on a set of 400,000 reactions extracted from US patents and their approach bypasses reaction templates by learning a reaction center identifier. In WLN, organic molecules are considered as a graph G = (V, E), where V is the set of atoms (vertices) and E is the set of associated bonds (edges), and a chemical reaction is a pair of molecular graphs (G_r_, G_p_). Thus, a reaction center is defined as a minimal set of graph edits needed (change of bond type for certain atom pairs) to transform reactant graph to product graph. The WLN will give every node a vector by training it with the information of all the neighbor nodes, which captures the local chemical environment of the atom and involves a comparison against a learned set of reference environments. Then with the local or global information (taking important reagent into account), they trained the model to predict reactivity label. After generation of candidates according to the reactivity label, they trained another Weisfeiler–Lehman Difference Network (WLDN) to rank the candidates. Their method achieved a top-1 accuracy of 74.0% on a test set of 40,000 reactions. Jin et al. claimed to outperform template-based approaches by a margin of 10% after augmenting the model with the unknown products of the initial prediction to have a product coverage of 100% on the test set. Differing from methods summarized in section Two-Step Models—Combination of Rule-based Model and Machine Learning, this approach is not only end-to-end, but also gets rid of the dependence on reaction rules. Though it definitely undergoes an intermediate step of reaction center (defined with certain data structure), this method is more “computational” than “chemical,” and the final model becomes more abstract than before.

**Figure 4:**
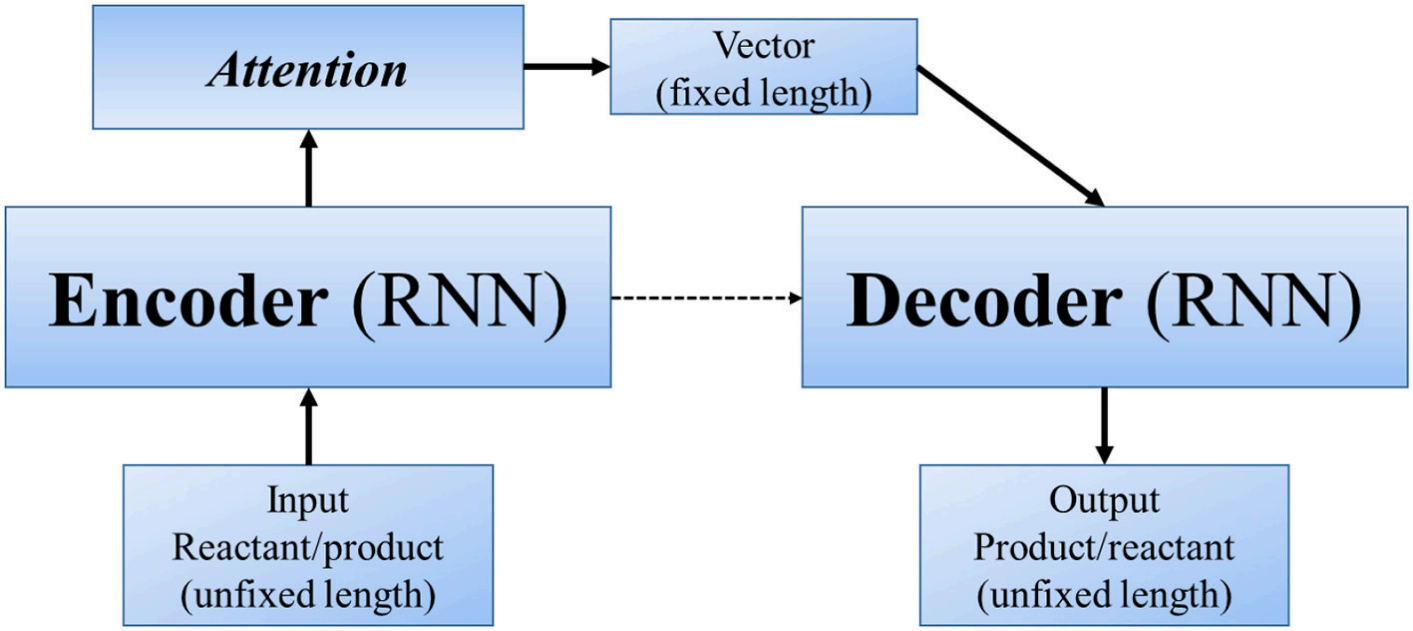
A schematic diagram of seq2seq—RNN with LSTM.

Other end-to-end methods can even skip the step of “reaction center” (or similar concepts). In Nam and Kim (2016) first applied seq2seq approach to reaction prediction. Seq2seq (Sutskever et al., 2014) is an algorithm using a multilayered Long Short-Term Memory (LSTM) to an input sequence (of unfixed length), and then another deep LSTM to decode a target sequence (also of unfixed length) from the vector (Figure 4). It was designed for translation between English and French, with the advantage that we only need to input large amount of parallel data, and the powerful deep neural network will automatically extract information and features of different languages and finally realize the translation. Molecule structures can be represented as linear SMILES strings, which can be decomposed to a list of atoms, bonds and several kinds of symbols. Hence, in a linguistic perspective, SMILES can be regarded as a language with grammatical specifications. In this sense, the problem of predicting products can be regarded as a problem of translating “reactants and reagents” to “products.” Nam and Kim used reaction database collected from patents by Lowe (2012) and 2001–2013 USPTO. Their model was based on the TensorFlow translate model (v0.10.0) (Abadi et al., 2016), from which they took the default values for most of the hyperparameters. When testing with Wade problems, the accuracy ranges between 0.35 and 0.85 in different problem sets.

With more training data, seq2seq model can behave much better in the field of reaction prediction. Schwaller et al. from IBM Research, Zurich also published a seq2seq approach (Schwaller et al., 2017). They built on the idea of relating organic chemistry to a language and explore the application of state-of-the-art neural machine translation methods, which are seq2seq models. Besides Lowe's data, they used data extracted from US patents granted and applications dating from 1976 to September 2016 in addition. The portion of granted patents is made of 1,808,938 reactions, described with SMILES. They took only single product reactions, corresponding to 92% of the dataset, to have distinct prediction targets. The accuracy is 80.3% for top-1, 84.7% for top-2, 86.2% for top-3 and 87.5% for top-5.

Actually, retrosynthesis is the opposite of reaction prediction. Given a product molecule, the goal is to find possible reactants. So, if we reverse the reaction direction, seq2seq can also solve pathway design problems, and this algorithm was developed by Liu et al. (2017). They used a set of 50,000 reactions extracted and curated by Schneider et al. (2016) The accuracy is 34.1% for top-1, 56.5% for top-5, 62.0% for top-10, and 71.9% for top-50. An important difference between this and Schwaller et al.'s method is that they did not omit reactions with multiple reactants or products. Instead, adding a dot between separate SMILES string can deal with this kind of reaction. In their approach, the dataset was classified into 10 reaction classes, including heteroatom alkylation and arylation, acylation and related processes, etc. The dataset was split into training, validation and test datasets (8:1:1). The accuracies of different reaction classes were calculated separately. Reversed input can increase the accuracy of recurrent neural networks, so they also reversed all the SMILES strings before training. Compared with other rule-based algorithms (Law et al., 2009), seq2seq retrosynthetic analysis behave much better in protection and de-protection reactions, that is to say, this algorithm can judge whether to introduce a protection group to avoid side reactions. As for common bond connecting and breaking reactions, however, this retrosynthetic analysis program cannot outperform traditional rule-based reactions. Liu et al. summarized all the errors into three types. First, the model outputs invalid SMILES string, which means the data is not enough for computers to comprehend the grammar of SMILES. Second, some reaction rules are wrongly predicted. Third, the overall reaction is chemically plausible but different from the result of the test set—this means the accuracy is underestimated in some ways. It is partially because of the presence of multiple reaction sites in the target molecule that can be disconnected retrosynthetically, so multiple possible reactant sets are chemically plausible.

The accuracy of retrosynthesis prediction is much lower than reaction outcome prediction. The difference between training and testing data is one reason, and multiple possible pathways for synthetic design is another reason. However, it is undeniable that none of the previous works can achieve end-to-end learning to the level of seq2seq models, and the accuracy of reaction product prediction has reached the highest level. An obvious disadvantage when compared to template-based methods is that the strings are not guaranteed to be a valid SMILES, which might decrease the prediction accuracy. Another limitation of the training procedure is multiple pathway choices. However, the problem of multiple choices only affects the apparent accuracy, and the algorithms can still give valuable results of retrosynthesis pathway predictions.

## Perspective

It is now clear that high-quality synthesis analysis systems are required to meet various needs in chemistry. With the development of learning algorithms and database, these needs are gradually being met or are the subject of active researches, but there are still many challenges to be overcome, including regiochemistry and stereochemistry. Computational chemical synthesis analysis and pathway design prediction is a task full of contradictions—more reaction rules mean more matches in each query, but are also likely to produce implausible examples; local scoring functions (for each step) may not give the best pathway, but designing functions emphasizing global minimum is so difficult. That's why recently scientists are shifting their attention to deep learning algorithm, however, methods like seq2seq are still not good enough for academic or commercial usage.

In an organic chemist's view, synthesis design is a kind of art rather than science—which intermediate, whether to protect… But for computer algorithms, whether rule-based methods or deep neural networks mainly focus on the availability of each step (some new methods could even solve the problem of the first step), and neglect the idea of “designing.” To reach the level of intelligent design, algorithms other than seq2seq and datasets which contain multiple-step synthetic data should be developed. If we regard chemical space as a “compound surface,” present methods are ready to tell us “how to take a correct step,” but we need the result of “shortest trajectory,” which is on a higher level.

Besides developing more methods for common chemical reactions, there are other fields needing the help of synthesis analysis. For example, biomimetic and biological synthesis is a tricky problem, and choosing proper enzymes can greatly reduce the complexity of synthesis pathway. Projects like PathPred (Moriya et al., 2010) used methods similar to database searching, but the result is limited due to the insufficient coverage of database and relatively poor ability of generalization. There are also learning-based methods like Dale et al.'s model (Dale et al., 2010) and rule-based methods like U Minnesota Pathway Prediction System (Gao et al., 2011) for biosynthesis pathway prediction. Predicting the condition of unknown reactions is also an extension of synthesis analysis systems.

In summary, in the past decades, there are plenty of exciting breakthroughs in chemical synthesis analysis and pathway design. Today, computers can be used to predict viable syntheses leading to quite complex targets and, with further development of computational methods, they can become better. As these systems of many varieties become more widely known and studied, the trend of chemical synthesis analysis systems will become more apparent and will stimulate research and development in directions not yet envisioned.

## Author contributions

All authors listed have made a substantial, direct and intellectual contribution to the work, and approved it for publication.

### Conflict of interest statement

The authors declare that the research was conducted in the absence of any commercial or financial relationships that could be construed as a potential conflict of interest.
